# A Novel Way to Grow Hemozoin-Like Crystals In Vitro and Its Use to Screen for Hemozoin Inhibiting Antimalarial Compounds

**DOI:** 10.1371/journal.pone.0041006

**Published:** 2012-07-18

**Authors:** Vincent Thomas, Ana Góis, Bruce Ritts, Peter Burke, Thomas Hänscheid, Gerald McDonnell

**Affiliations:** 1 STERIS SA R&D, Fontenay-aux-Roses, France; 2 Unidade de Microbiologia Molecular e Infecção, Instituto de Medicina Molecular, Faculdade de Medicina de Lisboa, Lisbon, Portugal; 3 STERIS Corporation, St. Louis, Missouri, United States of America; 4 STERIS Corporation, Mentor, Ohio, United States of America; 5 STERIS Limited, Basingstoke, United Kingdom; University of Maryland, United States of America

## Abstract

**Background:**

Hemozoin crystals are normally formed *in vivo* by *Plasmodium* parasites to detoxify free heme released after hemoglobin digestion during its intraerythrocytic stage. Inhibition of hemozoin formation by various drugs results in free heme concentration toxic for the parasites. As a consequence, *in vitro* assays have been developed to screen and select candidate antimalarial drugs based on their capacity to inhibit hemozoin formation. In this report we describe new ways to form hemozoin-like crystals that were incidentally discovered during research in the field of prion inactivation.

**Methods:**

We investigated the use of a new assay based on naturally occurring “self-replicating” particles and previously described as presenting resistance to decontamination comparable to prions. The nature of these particles was determined using electron microscopy, Maldi-Tof analysis and X-ray diffraction. They were compared to synthetic hemozoin and to hemozoin obtained from *Plasmodium falciparum*. We then used the assay to evaluate the capacity of various antimalarial and anti-prion compounds to inhibit “self-replication” (crystallisation) of these particles.

**Results:**

We identified these particles as being similar to ferriprotoporphyrin IX crystal and confirmed the ability of these particles to serve as nuclei for growth of new hemozoin-like crystals (HLC). HLC are morphologically similar to natural and synthetic hemozoin. Growth of HLC in a simple assay format confirmed inhibition by quinolines antimalarials at potencies described in the literature. Interestingly, artemisinins and tetracyclines also seemed to inhibit HLC growth.

**Conclusions:**

The described HLC assay is simple and easy to perform and may have the potential to be used as an additional tool to screen antimalarial drugs for their hemozoin inhibiting activity. As already described by others, drugs that inhibit hemozoin crystal formation have also the potential to inhibit misfolded proteins assemblies formation.

## Introduction

An essential part of malaria control is effective treatment and drug resistance is threatening these efforts [1]. Thus, new antimalarial drugs have become a research priority and hemozoin (malaria pigment) inhibition is a very attractive target because it appears to be an immutable pathway. In fact, the classical quinoline antimalarials are all thought to act by inhibiting hemozoin crystal formation, a pathway used by the *Plasmodium* spp. to detoxify free heme molecules after hemoglobin digestion [2].

Synthetic hemozoin (sHz), also called β-hematin, is structurally identical to hemozoin [3] and can be produced by chemical synthesis using hemin [4]. This was adapted in diverse assay formats to measure the hemozoin inhibition of drugs and new compounds [5], [6], [7], [8], [9], [10], [11]. However, it has been difficult to standardize these assays and even results are not consistent between all assays. For example, in one assay primaquine inhibited hemozoin formation [7], while in another it did not [11]. Certainly this could be explained by the various assay conditions, different reagents, pH and incubation times, or different types of initiators [5], [6], [7], [8], [9], [10], [11]. Furthermore, some assays use rather toxic reagents like pyridine [6], [10] or isotopes [11], while many depend on a complex readout: the produced hemozoin is washed, transformed back into heme and then read spectrophotometrically [7], [10]. Interestingly, quinoline derivatives and cyclins present anti-prion activity *in vitro* [12], [13], [14]. These observations and similar structure−activity relationships have been proposed for the anti-prion activity of quinoline derivatives [15]. In prion disease the aggregation of physiological proteins leads to fibrillar precipitates, although neither the initial seeding mechanism trigger nor the origin of the nucleation are clearly understood. Initially, we set out to find a novel and simple replacement assay for the currently used complex, time intensive and expensive *in vivo* and *in vitro* assays to evaluate the therapeutic and decontamination efficacy against transmissible misfolded proteins (prion diseases). We investigated a “replicating agent”, described in the 1980s, and termed ”Ileal Fluid Dependent Organism” (IFDO) which, interestingly, presented susceptibility to inactivation treatments comparable to prions: resistant to steam and aldehydes but inactivated by guanidine thiocyanate and sodium hydroxide [16], [17], [18], [19].

However, despite these intriguing results, the exact nature of “IFDO” remained unknown. In this study we describe “IFDO” as being hemozoin-like crystals (HLC), similar to synthetic hemozoin crystals (β-hematin) and hemozoin crystals obtained from *Plasmodium falciparum* cultures. Growth of these novel HLC requires few reagents and occurs at physiological pH (7) at 37°C. We present data on the potential use of the growth of these novel HLC as a novel and simple inhibition assay to screen for antimalarial drugs.

## Results

### Growth of New Hemozoin-like Crystals (HLC)

**Figure 1:**
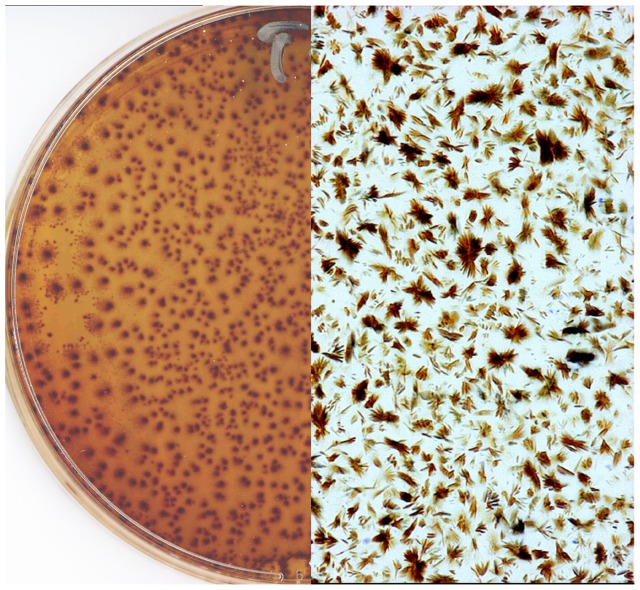
Growth of hemozoin-like crystals on agar plates. Crystal appearance after 7 days growth on Mycoplasma agar plates supplemented with horse blood. Right panel: magnification ×400.

**Figure 2:**
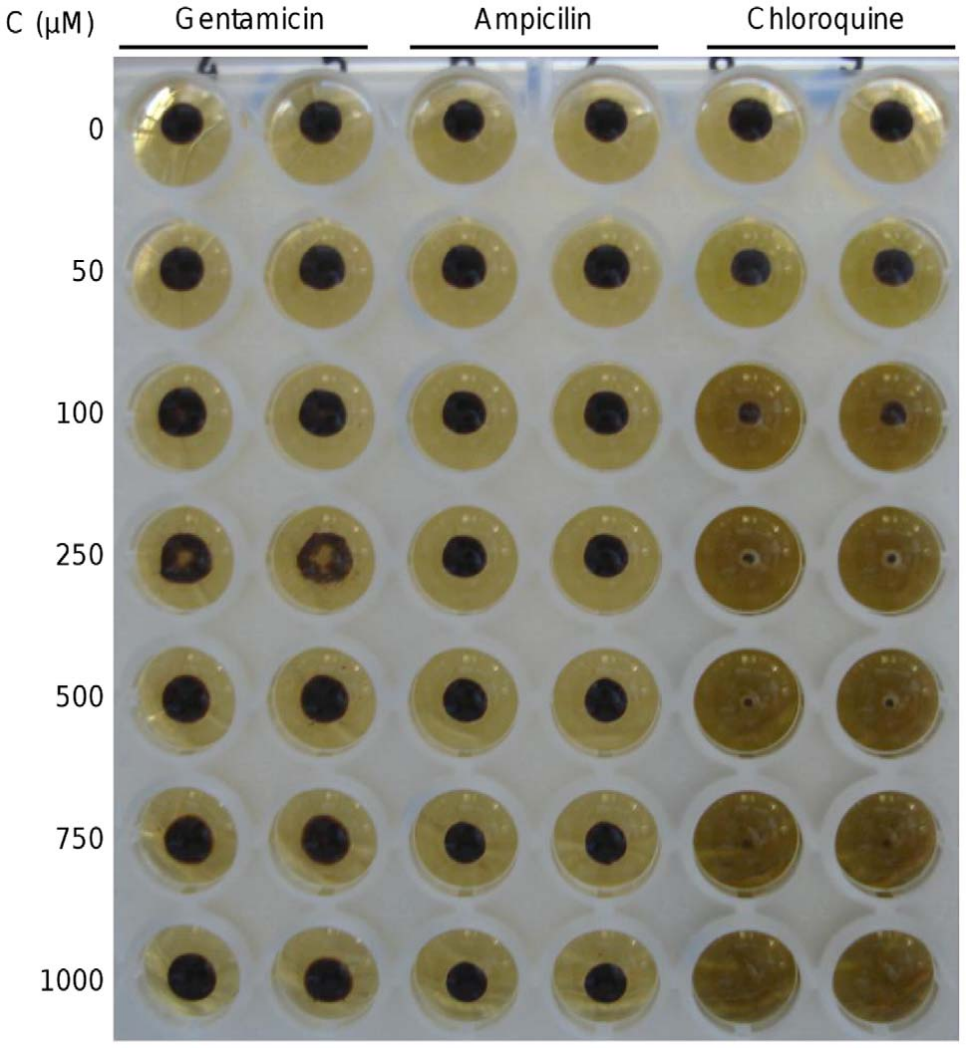
Growth of hemozoin-like crystals in broth (microtiter plate). A dark precipitate is easily visible with the naked eye. For the drug inhibition assay reported in Table 3, the first well with less growth than the drug free control was read as negative. Gentamicin and ampicillin show no inhibitory effect even at 1 mM, while chloroquine inhibits HLC growth already at 100 µM concentration.

As previously described [16], a “bacterial colony like” growth was observed following seeding of cultures in broth or on surface agar with “IFDO”. Structures resembling bacterial/fungal ‘colonies’ appeared on plates after 7 to 10 days of incubation (Figure 1) and there was a correlation between optical density of initial inocula and the number of colonies observed. A brown-black pellet progressively appeared in seeded broths (Figure 2) and optical density stabilized after 5 to 7 days of incubation. The results of different assay conditions are shown in Table 1. Interestingly, replacing lysed blood with commercial hemin resulted in a somewhat reduced growth. Seeding is essential for the growth to occur, but using sHz was equally effective as using “IFDO”. Lowering the pH reduced the growth, as did exposure to ambient atmosphere, the use of a different culture broth or replacing horse serum by human serum. Broth without pancreatin did not allow pellet formation.

**Table 1:** Hemozoin-like crystals growth conditions.

Conditions changed	Growth	Comments
Basic condition*	+++	Black deposit accumulation, visible from 3^rd^ day of incubation, stabilizing after 5–7 days; smaller black deposit forms in some of the non-seeded wells; medium turns brownishin the second day of incubation
Lysed horse blood extract(instead of human blood)	++++	Despite the higher yield, it does not grow faster/growth is not detected earlier
Hemin (instead of human blood)	+	Growth occurs in a lesser extent and slower
Human serum (instead of horse serum)	++	Growth occurs in a lesser extent and slower
Brain Heart Infusion broth (instead of Mycoplasma broth)	+/−	Small black deposits form as in non-seeded normal assay condition (first row)
pH 5 (instead of pH7)	++	More diffuse growth
Ambient atmosphere (instead of 5% CO_2_)	++	Growth occurs in a lesser extent and slower
Seeding with synthetic hemozoin (instead of “IFDO”)	+++	Growth identical to IFDO seeding
Without pancreatin	−	No black deposit formation; medium stays reddish and does not turn brownish in the second day of incubation

* Basic assay conditions are: 7 days incubation; Mycoplasma broth base, Tween 80, horse serum, 10% pancreatin, water, with lysed human blood extract; 37°C, 5% CO_2_, pH 7.

Production of sHz from hemin and purification of nHz from *P. falciparum* cultures was effectively done by our group as previously described [20].

### Transmission and Scanning Electron Microscopy

**Figure 3:**
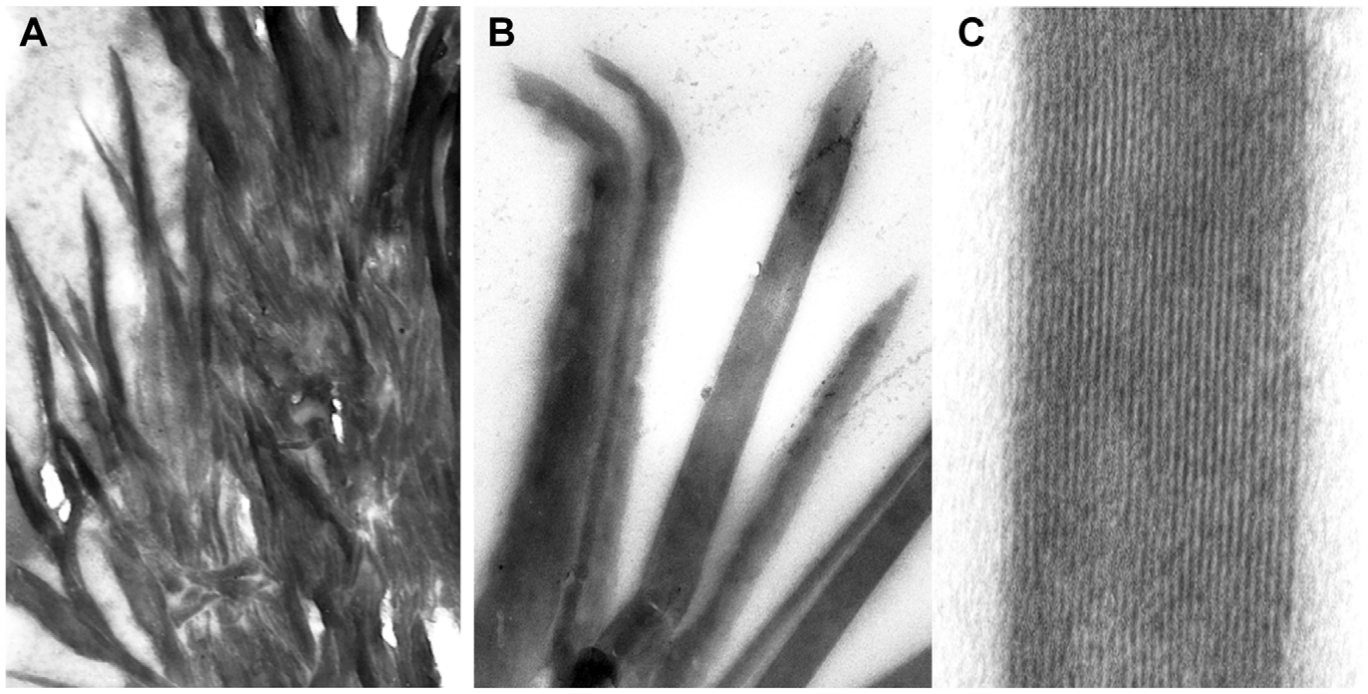
Transmission electron microscopy of hemozoin-like crystals. TEM images of hemozoin-like crystals reveal needle-like particles with a fibrillar aspect. Magnifications: X 28 000 (A), X 75 000 (B), X 155 000 (C).

**Figure 4:**
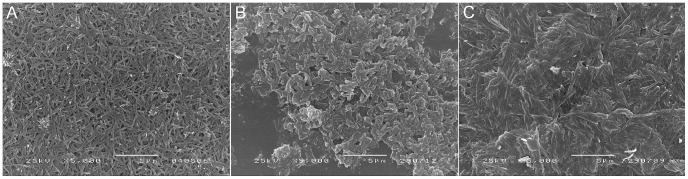
Scanning electron microscopy of synthetic and natural hemozoin and hemozoin-like crystals. SEM images of synthetic hemozoin (a), natural hemozoin (b) and hemozoin-like crystals (c) reveal crystalline needle-like particles with an almost identical shape and size.

Transmission electron microscopy of the pellet resulting from the growth confirmed a needle-like structure (Figure 3). Scanning electron microscopy shows a crystal-like structure with protruding needles (Figure 4) with an approximate size of 2 µm. Images are very similar to those obtained from sHz or nHz (Figure 4a and 4b). The structures were thus designated hemozoin-like crystals (HLC).

### Analysis and Comparison of New Hemozoin-like Crystals with nHz and sHz

Hemozoin-like crystals (HLC) are very similar to nHz and sHz when observed with light microscopy, although they show a higher tendency to aggregate in suspension. HLC are stable and do not dissolve in water, ethanol, methanol or DMSO. HLC also depolarize like nHz and sHz, using polarizing microscopy. Similarities observed using SEM are described above (Figure 4).

No proteins could be detected from different preparations of the HLC, including pre-sonicated preparations. Various conditions were tested to adapt the protein quantification kit but all failed to detect any significant protein. Western-blot analysis using Coomassie blue or silver nitrate staining failed to detect any specific associated protein. We also used 20% SDS gels to detect low molecular weight proteins but here again no specific proteins or small peptides could be consistently detected.

**Figure 5:**
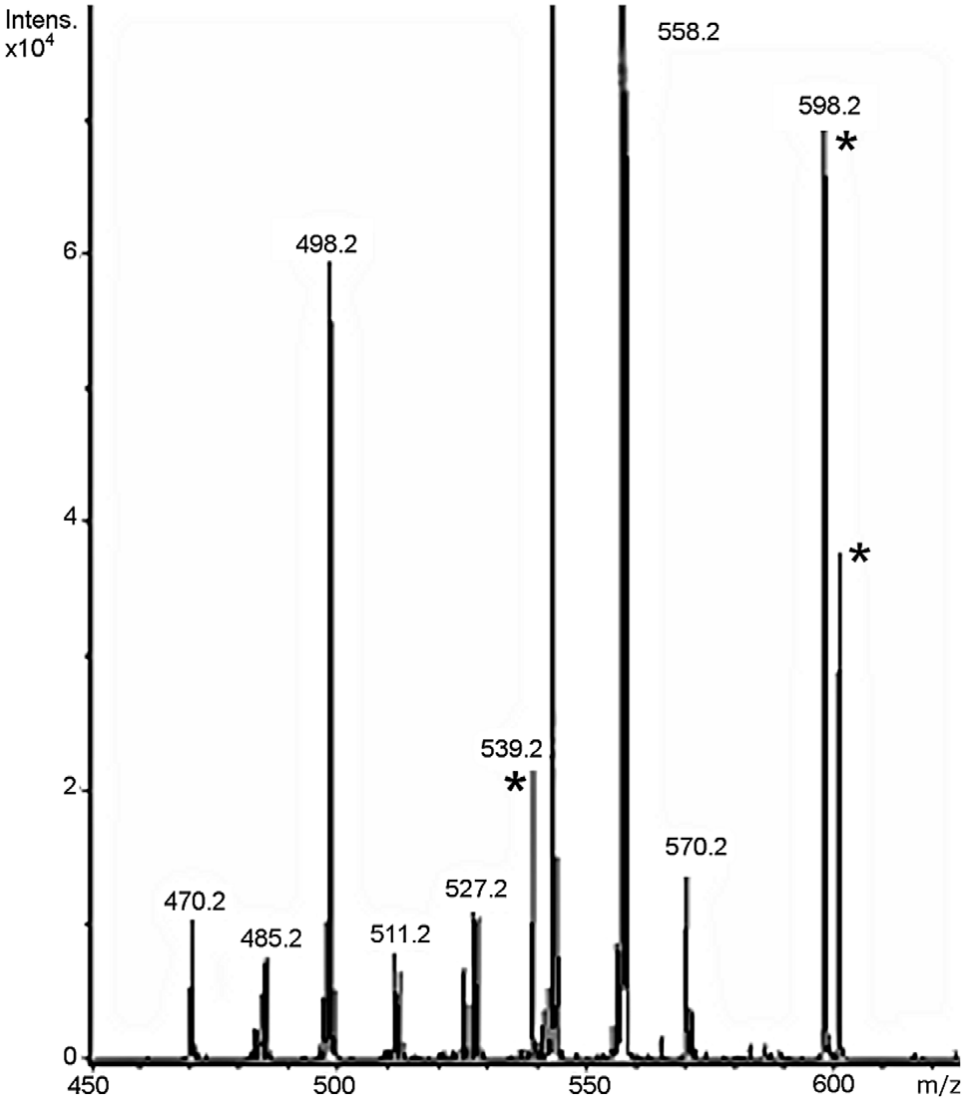
Maldi-Tof analysis of hemozoin-like crystals. Laser desorption mass spectrum of purified hemozoin-like crystals sample grown in modified Mycoplasma broth. Eight of 11 fragmentation peaks were identical to peaks previously reported when fragmenting ferriprotoporphyrin IX [21]. Additional peaks are indicated by a star. The ion at 598 may represent rearrangement and loss of water from the parent ion at 616 Da. It is interesting to note that the ion at 539.2 also represent loss of water from the 557 ion. The ion at 602 Da, if originating from the parent ion, would represent loss of a methylene (-CH2-) group.

Using MS analysis, the hemozoin-like sample yielded a unique intense ion at 616 Da when compared to controls. Analysis of this ion provided evidence for the presence of ferriprotoporphyrin IX (FP) materials similar to that observed by Demirev *et al.* [21]. Fragmenting this ion using direct infusion MS/MS resulted in a spectrum nearly identical to that observed when fragmenting FP (Figure 5); the supplementary ion at 598 Da could result from loss of water from the parent ion or the addition of an ACN molecule to the peak at 557 Da. The UHPLC analysis gave very similar chromatograms. No other associated structures were detected.

Heme contamination, as assessed by thin layer chromatography, was in the order of 37%, as compared to <1% observed in nHz or sHz preparation. Several attempts to reduce this contamination rate by extensive washes with water or methanol failed to reduce this figure.

**Figure 6:**
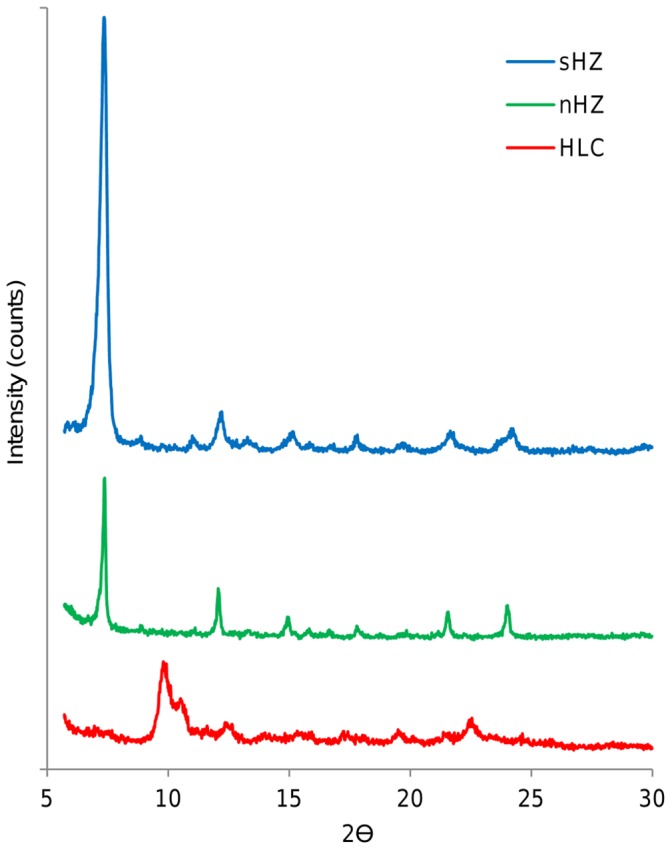
X-ray diffraction patterns of synthetic and natural hemozoin and hemozoin-like crystals. X-ray diffraction patterns of synthetic hemozoin (blue), natural hemozoin (green) and hemozoin-like crystals (red). The curves of natural and synthetic hemozoin are identical, while the curve of HLC shows additional peaks confirming that HLC are not identical to sHZ or nHz.

X-ray diffraction showed identical results for nHz and sHz (Figure 6). The results for HLC showed several additional peaks (Figure 6), confirming that HLC are indeed not identical to sHz or nHz.

### Hemozoin-like Crystal Growth Inhibition by Antimalarial and Antibacterial Compounds

In a first set of screening experiments, direct microscopic observation of HLC growth allowed visualization of numerous, needle-like particles. None of the wells showed cultivable contamination from bacteria or fungi. The results are summarized in Table 2. All quinoline derivatives and, interestingly, tetracyclines inhibited the formation of HLC at 1 mM. Chloroquine and polymyxin B were also effective at lower concentrations (100 µM), but none of these compounds inhibited crystal formation at 10 µM. The sulphated polyanions DS-500, PPS and CR36 did not display any inhibition effect at tested concentrations (Table 2).

**Table 2:** Inhibition of hemozoin-like crystals formation by several molecules at various concentrations in Mycoplasma broth.

	Compounds concentrations			
	1 mM	100 µM	10 µM	1 µM
Chloroquine	−	−	+	+
Doxycycline	−	+	+	+
Quinacrine	−	+	+	+
Quinine	−	+	+	+
Tetracycline	−	+	+	+
Rolitetracycline	−	+	+	+
Polymyxin B	na	−	+	+
CR36	+	+	+	+
Dextran sulphate 500	+	+	+	+
Pentosan polysulfate	+	+	+	+

“−” means no growth, “+” means visible growth.

na: not applicable due to non-HLC precipitates impairing observation.

In a second set of experiments, several antimalarial and antibacterial compounds were tested over a larger range of concentrations. The results are summarized in Table 3. Chloroquine and amodiaquine showed the strongest HLC growth inhibition (100 µM) followed by quinine, quinacrine, halofantrine (250 µM), and mefloquine (500 µM). Interestingly, artemisinin also inhibited HLC growth at 250 µM concentration. As expected, antibacterials, namely gentamicin, ampicillin and clindamycin showed no inhibitory effect at all.

**Table 3:** Inhibition of hemozoin-like crystal formation by several antimalarials and antibacterial agents.

	Compounds concentrations (µM)						
	1000	750	500	250	100	50	0
Chloroquine	−	−	−	−	−	+	+
Amodiaquine	−	−	−	−	−	+	+
Mefloquine	−	−	−	+	+	+	+
Quinine	−	−	−	−	+	+	+
Quinacrine	−	−	−	−	+	+	+
Halofantrine	−	−	−	+	+	+	+
Artemisinin	−	−	−	−	+	+	+
Clindamycin	+	+	+	+	+	+	+
Gentamicin	+	+	+	+	+	+	+
Ampicillin	+	+	+	+	+	+	+

“−” means no growth, “+” means visible growth.

A correlation was observed between processes (including steam sterilization and alkaline chemicals) previously shown to be effective against prions and HLC reduction. Interestingly, treatments reported as partially efficient against prions were only partially efficient or completely inefficient against HLC (Data S1).

## Discussion

In this study we set out to find a novel assay to test drugs as well as chemical and physical processes against prions because current gold standard methods are expensive and time-consuming [22]. With this perspective, we were interested to study a model described by Burdon *et al*. because it appears to share some similarities to prion agents since the, yet unidentified, “agent” forms self-propagating precipitates under well defined conditions [16], [18]. Moreover, precipitates can be broken into smaller size particles which can serve as nuclei for the generation of new aggregates. The nucleation phenomenon was described by Burdon *et al* as being inhibited by various physicochemical treatments in a way that compares to inhibition of prion propagation [16], [18] (Data S1).

However, the morphological analysis of these aggregates by optical, polarizing and transmission electron microscopy revealed similar structures to those seen with natural (nHz) or synthetic hemozoin (sHz). Especially scanning electron microscopy images are almost identical to those of nHz as observed by us (Figure 4) and described in the literature [4], [23]. Also, the dimension and size of the crystals appear identical. Our analysis identified the hemozoin-like crystals (HLC) as being ferriprotoporphyrin IX aggregates. No specific proteins or DNA could be detected from the HLC samples using protein quantification methods, western-blot analysis, silver nitrate staining and SDS-PAGE, which has also been described for sHz (ß-hematin) and purified nHz [4], [24].

However, X-ray diffraction data reveal that HLC are not identical to sHZ or nHz (Figure 6). Heme aggregates can form different bonds between the heme moieties, for example µ-oxo dimers or π-π dimers [2], [8]. Thus, it is possible that HLC are indeed not “true” hemozoin crystal aggregates (π-π dimers), but hemozoin like structures where the heme moieties are linked differently. Importantly, HLC present an average 37% remaining heme contamination, which could contribute to explain why it is different from hemozoin, in the sense that the crystals could be constituted by heme and ß-hematin. However, X-ray diffraction patterns of HLC and sHz are sufficiently different to indicate that HLC samples are unlikely to contain a phase that would show sHz specific peaks. It should be noted that most reports on hemozoin inhibition assays give little information on the methods used to identify the nature of the final aggregates [25], [26], 27,28. Some used only IR spectroscopy, while few employed scanning electron microscopy or X-ray diffraction [6], [29], [30], as in this study. Perhaps, it may be possible that the end product in some of these described assays was also not “true” hemozoin, yet it allowed to establish the hemozoin inhibitory effect of antimalarial compounds. In fact, it could be argued that it is less important that the final end product is “true” hemozoin, as long as the assay detects hemozoin inhibitors reliably. Of note, HLC are stable enough to not dissolve in DMSO, which is used to remove unreacted hematin in the end of hemozoin inhibition assays to allow hemozoin quantitation, because it “can also dissolve heme aggregates, but not ß-hematin crystals” [8].

The overall results of the HLC inhibition assay are consistent with previous results with 4-aminoquinolines, like chloroquine, showing the strongest inhibition, followed by the other quinoline drugs, like quinine or mefloquine [5], [6], [8]. On the other hand and as expected antibacterials like gentamicin, ampicillin or clindamycin showed no inhibitory effect. Interestingly, some authors report the strength with which drugs bind to heme, implying that the heme-drug adduct is the major contributor to inhibiting Hz growth [31]. However, others argue that the binding of antimalarial drugs to the hemozoin crystal surface is inhibiting the further growth and that binding preferences to the different sites of the crystal (which grow at different rates) may even explain the different inhibitory effects of antimalarials [2]. Of note, the described HLC method uses crystals to seed the assay, while almost all other described assays use only heme or hematin [7], [8] and few use some initiator, like Tween 20 [5] or another detergent [10].

Recently, a strong case was made for the idea that the target of artemisinins is PfATP6/SERCA and that artemisinins do not act by inhibiting hemozoin in the parasite [32]. However, our data show that artemisinins inhibit HLC formation (Table 3), which is in keeping with previous other studies [7], [23], although some argued that the hemozoin inhibition occurs only with some assays [9] or that artemisinins only form heme-adducts under reductive conditions [31].

An unexpected finding was the inhibition of HLC in the presence of tetracycline and tetracycline derivatives (Table 1) because current opinion favours the idea that the specific target of these drugs is the apicoplast [33]. This explains the fact why the drug effect *in vitro* is mainly detectable in the second generation [34]. However, contrary to clindamycin, tetracyclines also have an effect during the first parasite cycle, which was explained by a possible inhibition of prokaryotic protein synthesis in the mitochondrion [34]. The preliminary results of the HLC inhibition may indicate that tetracyclines might also inhibit hemozoin formation which could contribute to parasite killing during the first cycle.

Several hemozoin inhibition assays have been described in the past [5], . In principle, the ß-hematin inhibition assay is based on *in vitro* crystallization of heme or hematin. Crystallization occurs during a defined time interval (1–18 h) at a certain temperature (37–60°C), after adding glacial acetic acid or acetate buffer at pH 5 to hemin chloride or hematin solution in DMSO or NaOH. To remove non-crystalline heme, the final product is washed with DMSO and then dissolved back into heme again using NaOH, and finally measured by spectrophotometric reading at A_405nm_ [8]. To assess if a drug or compound inhibits ß-hematin formation, it is included in the reaction and then the inhibition quantified as the decrease in final ß-hematin concentration. As an alternative to transforming ß-hematin back into heme, colorimetric assays use pyridine to coordinate with unreacted hematin and the orange-pink color formed allows visual as well as spectrophotometric identification of ß-hematin inhibition [6].

However, several aspects of these assays deserve comment: (i) the use of highly concentrated solutions of both hemin (final concentration of 1–2 mM) and drugs (1–20 molar equivalents relative to hemin), as well as acid (8–12.9 M); (ii) multiple manipulation steps in the procedure (pipetting, washing steps); (iii) centrifuging microtiter plates at 3300 *g*, which may not be available in some laboratories because existing centrifuges may not have the necessary rotors to achieve such a force; and (iv) an often complex measurement of the end product, involving transformation of hemozoin back into heme, or using pyridine [6], which has significant toxicity (category 4 of acute toxicity [35]).

Contrary to this, the HLC inhibition assay is easy to set up. It requires rather basic and inexpensive reagents, at low concentrations, and only needs very simple laboratory infrastructures. Once set up, there are no further manipulation steps. Also, the final read-out is very simple, as it is based on visual observation of the end product.

Analogies between the *in vitro* activities of therapeutic compounds against hemozoin and various misfolded proteins is supported in other studies, leading to the hypothesis that therapeutic compounds presenting activity against the malaria parasite could theoretically also have a benefit in Creutzfeldt-Jakob and/or Alzheimer diseases [36], [37]. Klingenstein *et al* screened a series of quinolines derivatives for their ability to inhibit *Plasmodium* parasite growth, and then subsequently screened the same compounds for ability to cure a persistently prion infected cell line [15]. They observed similar structure-activity relationships between antimalarial and anti-prion drugs and concluded that some molecular targets of antiprion and antimalarial substances overlap [15]. The link between antimalarial and anti-prion activity has been confirmed by studies with other classes of compounds [14]. Sulphated polyanions have been investigated as potential drugs against prion. They are supposed to act indirectly by competing with endogenous heparan sulfate proteoglycans as co-receptors for the prion protein (PrP) on the cell surface [38]. This fundamental difference explains why sulphated polyanions did not display any inhibitory effect against HLC growth in our assay. Concerning decontamination treatments, the analogy between abrogation of the nucleation phenomenon for hemozoin and abrogation of infectivity for prion diseases has been reported by Burdon et al and was confirmed in our study (Data S1). It is somewhat more difficult to understand and would require additional investigations to better describe the exact effect of decontamination treatments on HLC.

As a conclusion, it seems that HLC can be grown using a simple *in vitro* assay and under basic laboratory conditions. Although HLC are not identical to sHz, the results of the inhibitory effects of the quinoline drugs, including their different potency, are consistent with those reported in the literature. However, the HLC assay indicates that both artemisinins and tetracyclines inhibit HLC production, results which warrant further studies.

## Methods

### Growth of Hemozoin-like Crystals

Original “IFDO” cultures were kindly provided by Dr D. Burdon. Culture methods were adapted from the original protocol [16]. The broth medium was prepared by autoclaving 35.5 g of Mycoplasma Broth Base (Oxoid CM403) in 950 ml distilled water and 2 ml Tween 80. After cooling at 50°C, 1.33 ml horse serum (Gibco) and 20 ml of a 10% pancreatin (Sigma) solution in phosphate-buffered saline (PBS) (Gibco) were added. 30 ml of a fresh blood extract was added and prepared by (i) washing fresh defibrinated horse blood (Oxoid) 3 times in PBS, (ii) lysing red cells by adding an equal amount of sterile distilled water, (iii) submitting red cells to 3 successive freeze-thaw cycles and 5 minutes sonication, (iv) centrifuging for 15 minutes at 3500 rpm and discarding the pellet. The complete medium was filtered at 0.22 µm and immediately used.

Alternatively, in Lisbon, red blood cells from healthy human donors (obtained from a “buffy coat” from the Instituto Português do Sangue) were used to prepare the fresh blood extract. A modified broth medium was also developed, replacing blood extract by 50 µM hemin (Fluka 51280, stock solution prepared in NaOH 0.4 N and 0.22 µm-filtered); pH of the medium was adjusted at 7.2 before filtering at 0.22 µm. The agar medium was prepared with Mycoplasma Agar Base (Oxoid CM401). Cultures were prepared by seeding 50 µl of a McFarland 6 “IFDO” crystal particles suspension in 10 ml of broth or onto plates, and incubating at 37°C and 5% CO_2_ for 5 to 7 days. Besides these, other growth conditions were tested, including: adjusting the medium to pH 5; incubation at ambient atmosphere instead of 5% CO_2_; using Brain Heart Infusion broth instead of Mycoplasma broth; replacing horse serum by human serum (obtained from a healthy donor); excluding pancreatin from the medium; and seeding with sHz instead of “IFDO”.

### Synthetic Hemozoin Production

Synthetic hemozoin was obtained by the method described by Slater *et al.* (1991) [39], with some modifications. Briefly, 475 mg of hemin chloride (Fluka) were dissolved in 100 ml of 0.1 N NaOH and heme was precipitated by slowly adding 35 ml of glacial acetic acid. Crystallisation was promoted by overnight incubation of the mixture at 80°C. Non-crystalline heme was then removed by washing three times with 1 vol. of 100 mM sodium bicarbonate (pH 9.1) during 3 hours, centrifuging for 15 minutes at top speed. The pellet was further washed three more times in ultrapure water (MilliQ Synthesis) and finally ressuspended in 5 vol. of ultrapure water, quantified as heme-equivalents, after solubilization in 20 mM NaOH for 1 hour, using QuantiChrom™ Heme Assay Kit (Hayward CA, USA), and stored at 4°C.

### *Plasmodium falciparum* Hemozoin Purification

*P. falciparum* hemozoin was purified after saponin harvesting of parasites from 1 l of *P. falciparum* (3D7 strain) cultures at 1% hematocrit, enriched in trophozoites at a parasitemia of at least 10%, as previously described by Coban *et al*. (2002) [40]. In short, parasites were extensively washed with PBS, pellet was sonicated for 5 minutes, extensively washed with 2% sodium dodecyl sulfate (SDS) and then incubated overnight with 2 mg/ml Proteinase K. After being washed with 2% SDS again, the pellet was incubated for 3 hours in 6 M urea and then washed with 2% SDS and ultrapure water. Purified *P. falciparum* hemozoin was ressuspended in ultrapure water, quantified and stored at 4°C as for synthetic hemozoin.

### Heme Contamination Assessment

Hemozoin and hemozoin-like crystals produced were assessed for remaining heme contamination using thin layer chromatography. Along with hemin chloride solutions of known concentrations, 10 microliters of sample, previously diluted in methanol to the higher concentration of hemin used, is eluted on a silica gel plate inside a methanol-saturated tank for 30 to 40 minutes. The result is analyzed by determination of the integrated densities on the plate, using ImageJ software and calculation of remaining heme contamination percentage in the sample.

### Transmission Electron Microscopy

Hemozoin-like crystals were produced in Mycoplasma broth, then concentrated by centrifugation at 3 000 rpm for 5 minutes, washed in PBS, fixed for 3 hours at 4°C in 4% glutaraldehyde, then with 1% osmium tetroxide for 1 hour at room temperature. Dehydration was performed by successive washes in increasing acetone concentrations (50–100%). Samples were incubated for 1 hour in a vol/vol suspension of acetone-epon and overnight in epon. They were then embedded in an epoxy resine (Fluka). Thin sections were cut from embedded blocks by a LKB 2088 ultrotome, deposited on copper grids coated with formvar (Sigma-Aldrich) and stained for 10 minutes with a solution of methanol-uranyl acetate and lead nitrate with sodium citrate in water. Grids were examined with a transmission electron microscope (PHILIPS EM 201 C, Philips, Eindhoven, the Netherlands).

### Scanning Electron Microscopy

Ten microliters of hemozoin and hemozoin-like crystal samples were allowed to dry overnight in air on top of a carbon tape on a metallic sample holder and were metalized for 30 minutes using JEOL, JFC-1200 with a gold target. Scanning electron microscopy was performed with JEOL, JSM-2500 LV scanning electron microscope.

### Analysis and Comparison of New Hemozoin-like Crystal

A Protein Quantitation kit (MicroBC Assay Protein Quantitation Kit, Interchim) was used to detect putative proteins from HLC preparations. Briefly, particles grown in Mycoplasma broth were pelleted by centrifugation at 3 000 rpm for 5 minutes, washed once with PBS, centrifuged again, re-suspended in 1 ml lysis buffer and left for 20 minutes at 4°C. The suspension was then centrifuged at 14 000 rpm for 1 minute, the supernatant was collected and the pellet was re-suspended in 500 µl lysis buffer. Two fold dilutions of the supernatant and the pellet were used for protein quantitation.

Hemozoin-like crystals grown in 45 ml of Mycoplasma broth were used for Western-blot studies. They were washed twice with sterile PBS and the pellet was re-suspended in 400 µl of Laemmli buffer. The sample was then boiled for 5 minutes at 100°C before loading on a 14% SDS-PAGE gel. Migration was performed at 100 V for 2 hours. The gel was then transferred for staining with Coomassie blue or silver nitrate (ProteoSilver™ Plus Silver Stain Kit, Sigma).

Hemozoin and HLC were subjected to X-ray diffraction analysis. Crystals were re-suspended in 10 µl of absolute ethanol and allowed to dry in air on the silicon sample holders. X-ray diffraction pattern was then acquired with the X-ray diffractometer X’ Pert PRO in the 2θ range 5–30° with Cu Kα radiation (λ = 1,540562 Å), operating at 30 mA and 40 kV, with a step size of 0.0170° and scan step time of 100 seconds.

For Mass Spectrometry (MS) analysis, the new hemozoin-like particles were grown for 5 days in 50 ml Mycoplasma broth. The pellet was washed in sterile de-ionized water and inactivated by heating at 95°C for 30 minutes in 6M Guanidine-HLC. The suspension was then filtered at 0.45 µm and samples were concentrated with ZipTip C18 pipette tips (Millipore). The tips were washed with acetonitrile (ACN) and equilibrated with 0.1% trifluoroacetic acid (TFA). Analytes were adsorbed onto the C18 matrix directly from the HLC-Guanidine treated samples and tips were washed with 0.1% TFA. 10 to 100 µL of 50% ACN/water to 100% ACN with 0.1% TFA were used for elution.

For exact mass measurements, eluants were aspirated into the electrospray interface of a Waters LCT Premier aoTOF MS (probe voltage: 3000 V, collected mass range: 300–2000 Da). UHPLC separation was also performed using a Waters ACQUITY UPLC® equipped with an ACQUITY C8 column. An ACN/0.1% aqueous formic acid gradient was prepared starting with 25% ACN and ending with 97% ACN after 10 minutes (flow rate: 0.5 mL/min); eluant was split 1∶1 before entering aoTOFms (collected mass range: 100 to 1000 Da). An HCT Ultra (Bruker, Germany) was used for MS/MS analysis with direct infusion; two minutes isolation/fragmentation cycles were manually performed with the peaks identified above.

### Hemozoin-like Crystals Growth Inhibition by Antimalarial and Antibacterial Compounds

McFarland 6 “IFDO” suspensions in PBS were sonicated for 3 minutes and diluted 1/100 in fresh “IFDO” broth; 180 µl were distributed in wells of a 96 wells plate. Various classes of drugs were tested, including quinoline derivatives (quinine, chloroquine and quinacrine), tetracyclines (tetracycline, rolitetracycline and doxycycline), sulphated polyanions (dextran sulphate 500, pentosan polysulfate and the heparan mimetic CR-36) and polymyxin B. Drugs were prepared at 10 mM in sterile deionised water for quinine, chloroquine, quinacrine, polymyxin B, dextran sulphate 500 (DS-500), pentosan polysulfate (PPS) and CR-36, in 70% ethanol for tetracycline, doxycycline and rolitetracycline; pH was adjusted at 7±0.2 and 0.22 µm-filtered solutions diluted at 1 mM, 100 µM and 10 µM final concentrations in 12 wells per concentration. Plates were incubated at 37°C +5% CO_2_ for 5 days, and then 5 µl of all crystal-positive wells were seeded onto TSA, Mycoplasma and BCYE agar plates to confirm absence of bacteria. Five microliters from each well were also seeded onto fresh Mycoplasma agar plates to observe any regrowth. All tests were performed in triplicate (Table 2).

A similar procedure was followed to test a larger range of drug concentrations (Table 3), in order to determine a minimum inhibitory concentration. Stock solutions of chloroquine diphosphate, amodiaquine dihydrochloride, quinine hydrochloride dihydrate, quinacrine dihydrochloride, clindamycin hydrochloride, gentamicin and ampicillin were prepared in sterile ultrapure water, and those of mefloquine hydrochloride, halofantrine hydrochloride and artemisinin were prepared in absolute methanol. After being adjusted to pH 7±0.2 and 0.22 µm-filtered, drug solutions were diluted in the seeded wells to a final concentration of 50, 100, 250, 500, 750 and 1000 µM. Each drug was tested in duplicate, and plates were observed daily for 5 days of incubation to determine presence or absence of HLC growth (Figure 2). All drugs were purchased from Sigma-Aldrich except for mefloquine which was from Roche.

## Supporting Information

Data S1 **Decontamination tests.**
(DOC)
