# Supplementary material for: A Novel Way to Grow Hemozoin-Like Crystals In Vitro and Its Use to Screen for Hemozoin Inhibiting Antimalarial Compounds
Source: PLoS One. 2012 Jul 18;7(7):e41006. doi: 10.1371/journal.pone.0041006 (PMC3399802; doi:10.1371/journal.pone.0041006)
Supplement: Data S1 — Decontamination tests. (DOC) [file pone.0041006.s001.doc]

**Supplementary data: Decontamination tests**

### Methods

McFarland 6 hemozoin-like crystal (HLC) suspensions in PBS were sonicated for 3 min, diluted 1/10 in product to be tested and incubated for the appropriate time (Supp Table 1). Heat treated samples were diluted in sterile distilled water before autoclaving. After treatment products were neutralized by 1/10 dilution in Mycoplasma broth without supplements + 3% glycin, and incubating for 5 min at room temperature. Residual crystals were detected by seeding 20 µl neutralized samples in 180 µl of Mycoplasma broth. Twelve wells were seeded for every dilution. Crystal growth was observed and the decimal reduction was calculated. All tests were performed in triplicate. Interferences of the neutralization buffer and neutralized products with crystal growth were controlled.

**Results**

Precipitates in positive controls developed progressively during incubation, reaching maximum density in approx. 3 to 5 days. We were able to detect crystal formation in diluted wells corresponding to an initial titre of approx. 10^10^ crystal-forming nuclei per ml. No growth was observed in non-seeded wells (negative controls).

Results with various decontamination treatments are provided in Supplementary Table 1 and compared to previous results with prions (as determined in infectivity studies ). A correlation was observed between those processes (including steam sterilization and alkaline chemicals) previously shown to be effective against prions and HLC reduction. Interestingly, treatments reported as partially efficient against prions were only partially efficient or completely inefficient against HLC.

**Discussion**

Results obtained with the HLC model and inactivation by decontamination treatments are strikingly comparable to those obtained with various prion strains *in vivo* . It was confirmed in this study that HLC were inactivated by 6M Guanidine-HLC and heating at 95 °C for 30 min (the treatment used before proteomic analysis), being similar to inactivation studies with prions. Autoclaving was only poorly efficient at 121 °C, and partially efficient at 134 °C, which again compares with previously published results for prion decontamination . Recommended methods for prion inactivation (exposure to 1N sodium hydroxide or to 20 000 ppm sodium hypochlorite for 1 h) were fully efficient, as were treatments with an alkaline cleaner (Hamo-100) and a phenolic disinfectant (Environ LpH). Interestingly, the activity of Hamo-100 against HLC was not only due to the alkaline pH of the solution since exposure to NaOH 0.1N as a control and at a higher pH resulted in less efficient inactivation. The peracetic acid based product (STERIS-20) demonstrated partial activity, which correlates with prion infectivity study results. An enzymatic treatment (Klenzyme) was inactive against HLC, whereas it has been reported to be partially active against prion *in vivo*. Absence of activity against HLC was expected since proteins were not detected in the crystal. However, this correlates well with the finding that this treatment appeared to result only in the physical removal of prion infectivity from a contaminated surface and had no direct inactivating effect on prions in suspension studies . Despite this correlation, the predictive nature of the hemozoin-like crystal *in vitro* model may be limited (similar to other *in vitro* methods for studying prion infectivity) and requires further investigation.

**References**

1. Fichet G, Comoy E, Duval C, Antloga K, Dehen C, et al. (2004) Novel methods for disinfection of prion-contaminated medical devices. Lancet 364: 521-526.

2. Fichet G, Comoy E, Dehen C, Challier L, Antloga K, et al. (2007) Investigations of a prion infectivity assay to evaluate methods of decontamination. J Microbiol Methods 70: 511-518.

3. Taylor DM, Fraser H, McConnell I, Brown DA, Brown KL, et al. (1994) Decontamination studies with the agents of bovine spongiform encephalopathy and scrapie. Arch Virol 139: 313-326.

4. Taylor DM, Fernie K, McConnell I, Steele PJ (1998) Observations on thermostable subpopulations of the unconventional agents that cause transmissible degenerative encephalopathies. Vet Microbiol 64: 33-38.

5. Taylor DM (1999) Inactivation of prions by physical and chemical means. J Hosp Infect 43 Suppl: S69-76.

6. Brown P, Rohwer RG, Gajdusek DC (1986) Newer data on the inactivation of scrapie virus or Creutzfeldt-Jakob disease virus in brain tissue. J Infect Dis 153: 1145-1148.

## Supplementary Table 1 - Comparison of log-_10_ reductions observed when applying several physical and chemical decontamination treatments to IFDOs suspensions and to stainless steel wires contaminated with prion-infected brain-homogenate

|  |  |  |  |  |  |  |
| --- | --- | --- | --- | --- | --- | --- |
| **Product tested** | **Concentration** | **pH** | **Temperature** | **Time** | **Log-_10_ reduction observed** | |
|  |  |  |  |  | **IFDOs** | **Prion*^a^*** |
|  |  |  |  |  |  |  |
| Steam sterilization | n.a. | 7.2 | 134°C | 18min | 3.6 ± 1.1 | 3 to > 5.6*^b^* |
|  | n.a. | 7.2 | 120°C | 21min | 1.0 ± 0.0 | ≤ 4.0*^c^* |
|  |  |  |  |  |  |  |
| Hamo-100 | 1.6% | 12.7 | 43°C | 15min | 6.4 ± 1.0 | > 5.6 |
|  |  |  |  |  |  |  |
| Klenzyme | 0.8% | 7.7 | 43°C | 5min | 0 | 3.5 |
|  |  |  |  |  |  |  |
| LpH | 5% | 1.7 | 20°C | 1h | > 7.0 | > 5.6 |
|  |  |  |  |  |  |  |
| Sodium hydroxide | 1N | 14.0 | 20°C | 1h | > 7.0 | > 5.6 |
|  | 0.1N | 13.1 | 43°C | 15min | 3.9 ± 0.1 | 5.0*^d^* |
|  | 0.01N | 11.8 | 43°C | 15min | 1.0 ± 0.0 | 0.0*^d^* |
|  |  |  |  |  |  |  |
| Sodium hypochlorite | 20 000 ppm | 12.1 | 20°C | 1h | > 7.0 | > 5.6 |
|  |  |  |  |  |  |  |
| STERIS-20 | 0.25% | 5.8 | 55°C | 12min | 5.0 ± 0.7 | 3.5 |
|  |  |  |  |  |  |  |

*^a^*Log reductions based on animal infectivity studies . The observed reductions were consistent between publications.

*^b^*Depending on prion strains tested, and if autoclaving was performed with or without immersion

*^c^*15min autoclaving

*^d^*These assays were performed at room temperature
